# Evaluation of the Reparative Effect of Sinomenine in an Acetaminophen-Induced Liver Injury Model

**DOI:** 10.3390/cimb46010059

**Published:** 2024-01-21

**Authors:** Ahmet Kayalı, Ejder Saylav Bora, Hüseyin Acar, Oytun Erbaş

**Affiliations:** 1 Department of Emergency Medicine, Faculty of Medicine, Izmir Katip Çelebi University, Izmir 35270, Turkey; ahmet.kayali083@gmail.com (A.K.); dracar@hotmail.com (H.A.); 2 Department of Emergency Medicine, Izmir Atatürk Research and Training Hospital, Izmir 35360, Turkey; 3 Department of Physiology, Faculty of Medicine, Demiroğlu Bilim University, Istanbul 34395, Turkey; oytunerbas2012@gmail.com

**Keywords:** drug-induced hepatotoxicity, Acetaminophen, Sinomenine, BMP-7

## Abstract

Due to its rising global prevalence, liver failure treatments are urgently needed. Sinomenine (SIN), an alkaloid from sinomenium acutum, is being studied for its liver-repair properties due to Acetaminophen (APAP) overdose. SIN’s effect on APAP-induced hepatotoxicity in rats was examined histologically and biochemically. Three groups of 30 adult male Wistar rats were created: control, APAP-only, and APAP + SIN. Histopathological and biochemical analyses were performed on liver samples after euthanasia. SIN is significantly protected against APAP damage. Compared to APAP-only, SIN reduced cellular injury and preserved hepatocellular architecture. The APAP + SIN Group had significantly lower ALT, MDA, and GSH levels, protecting against hepatocellular damage and oxidative stress. SIN also had dose-dependent antioxidant properties. When examining critical regulatory proteins, SIN partially restored Sirtuin 1 (SIRT1) levels. While BMP-7 levels were unaffected, histopathological evidence and hepatocyte damage percentages supported SIN’s liver-restorative effect. SIN protected and repaired rats’ livers from APAP-induced liver injury. This study suggests that SIN may treat acute liver damage, warranting further research into its long-term effects, optimal dosage, and clinical applications. These findings aid liver-related emergency department interventions and life-saving treatments.

## 1. Introduction

On a global scale, the number of deaths that are attributed to liver failure is growing, and the number of individuals who are waiting for a liver transplant is also growing daily [1]. It has been established through recent research that the unregulated consumption of a variety of pharmaceuticals and chemicals poses a potential threat to the liver’s overall health and stability. Individuals who are predisposed to experiencing adverse effects from drug administration have the potential to experience liver damage, regardless of whether the drugs are administered in excessive amounts or within the therapeutic ranges that are recommended [2].

Acetaminophen (APAP) is the predominant medication utilized globally for alleviating pain and reducing fever. Simultaneously, APAP can induce dose-dependent centrilobular hepatic necrosis, which is a frequent reason for hospital emergency department admissions and the most prevalent form of drug toxicity [3]. The suggested oral dose for adults is 325–650 mg every 4–6 h, with a maximum daily dose of 4 g [3,4]. While APAP is considered safe when taken at therapeutic doses, it can cause centrilobular hepatic necrosis at higher doses [3]. Davidson and Eastham initially reported cases of acute hepatotoxicity caused by APAP overdose in 1966 [5]. Due to a deep understanding of the mechanism behind APAP hepatotoxicity, N-acetylcysteine (NAC) was implemented in clinical settings to remove the harmful reactive metabolite. Administering NAC is an effective method for treating APAP overdose. However, this protective effect is only seen during the initial phase of APAP-induced liver damage. In the mouse model, NAC does not protect after 4 h following an APAP overdose. However, the therapeutic window for NAC intervention in patients extends up to 10 h after an APAP overdose. The lag time allows for an optimal treatment window with NAC. If administered within the initial 10 h of an overdose, NAC has the potential to avert the development of liver damage [6] entirely. Patients who do not receive NAC promptly experience significant liver damage, which may lead to the development of acute liver failure (ALF). Therefore, liver transplantation is the definitive therapy for patients with ALF.

Acute hepatotoxicity caused by APAP exhibits a pattern of a necroinflammatory injury. Liver injury is histologically characterized by centrilobular hepatic necrosis and a mild inflammatory infiltrate. From a biochemical perspective, cases of APAP intoxication exhibit significant increases in serum aminotransferase levels, specifically aspartate aminotransferase (AST) and alanine aminotransferase (ALT). In the rodent model, after being exposed to APAP, a significant alteration in the liver was observed, which involved the depletion of glycogen and the formation of vacuoles in the centrilobular hepatocytes within 2 h. At 3 h, nuclear alterations were detected in centrilobular hepatocytes, and condensed nuclei characterized the necrosis of individual cells. At 6 h, the complete necrosis of the centrilobular areas was observed [7].

Moreover, sterile inflammation and inflammasome activation are observed in both mice and humans following an overdose of APAP [3]. Furthermore, certain studies have indicated that APAP hepatotoxicity is linked to the programmed cell death of liver cells, known as hepatocytes. However, in contrast to the large number of necrotic cells, only a few apoptotic cells were found in the livers of mice that were given a toxic dose of APAP [8]. Most studies have shown that necrosis, rather than apoptosis, is the leading cause of cell death in hepatocytes during acute liver injury induced by APAP.

APAP is one of the most commonly used and one of the oldest painkillers, with its low side effects and safety [9]. In addition to its analgesic effect, it also has antipyretic properties. Although the safety range of APAP is vast, it increases reactive oxygen products (ROS) at toxic doses and impairs antioxidant protective capacity [10].

The toxic effect of APAPon the liver is initiated by P450 (CYP) conversion to N-acetyl-p-benzoquinonimine (NAPQI). NAPQI removal from the system occurs primarily by glutathione (GSH) binding. When GSH is depleted, NAPQI is thought to bind to cellular macromolecules and cause hepatotoxicity. However, the precise mechanism by which cellular toxicity occurs is still a subject of debate [11].

Sinomenine (SIN) is an alkaloid from the Chinese medicinal herb sinomenium acutum. SIN’s safety profile and potent anti-inflammatory and immune-regulatory properties have generated significant interest [12,13,14]. Presently, it has been transformed into a collection of Chinese-exclusive medications known as Zhengqing Fengtongning (ZQFTN) that are utilized for the treatment of RA and other autoimmune disorders in China. The study demonstrated that SIN [15] reduced the activity of specific cytokines and mediators, including TNF-α and IL-1β. The correlation between SIN’s anti-inflammatory impact and RA’s advancement has been noted in both cellular and animal models of arthritis. It has an extended tradition of use in managing inflammatory diseases [16,17]. Scientific research has demonstrated that purified SIN has a notable therapeutic effectiveness for individuals with rheumatoid arthritis [16,18]. Nevertheless, no comprehensive investigation has been conducted to evaluate the anti-inflammatory properties of SIN on cytokines in both cellular and animal models, nor to assess its therapeutic efficacy on cytokines in a clinical context. Moreover, some studies show the immunosuppressive and anti-inflammatory effect of Sinomenine [12,19] and, on the other hand, the protective effect of the liver in fulminant hepatitis [20].

SIRT 1 is the sirtuin that has been studied the most extensively. It is implicated in both alcoholic and non-alcoholic fatty liver diseases [21]. SIRT1 has advantageous functions in regulating lipid metabolism in the liver, the control of oxidative stress in the liver, and mediating inflammation in the liver by removing acetyl groups from certain transcriptional regulators, thus preventing the advancement of liver diseases [21].

A superfamily of bone morphogenetic proteins (BMPs) belongs to the transforming growth factor beta (TGFβ) family. Different tissues in the body, most notably the liver, are responsible for the production of BMP-7 [22]. Concerning the development and expansion of the liver, BMP-7 is a significant factor. Most of the time, the concentration of BMP-7 in the bloodstream falls within the normal range, typically between 100 and 300 pg/mL. However, adult hepatocytes do have receptors for BMP-7 even though the liver does not express BMP-7. As a result, they hypothesized that BMP-7 could act as an innate controller of the proliferation of adult hepatocytes and the maintenance of liver equilibrium14. It has been demonstrated in several studies that BMP-7 possesses antifibrotic properties in the liver [23,24].

The objective of this study is to assess the impact of SIN on the liver toxicity induced by APAP in rats using histologic and biochemical evidence.

## 2. Materials and Methods

### 2.1. Animals

A total of 30 adult male Wistar rats, with a weight range of 200–210 g, were utilized for the study. The animals were kept in boxes under controlled conditions, with a 12-h cycle of light and darkness at 22 ± 2 °C. The subjects were provided with a standard diet of pellets and had unrestricted access to tap water throughout the study. All chemicals were obtained from Sigma-Aldrich Inc., St. Louis, MO, USA. unless otherwise noted.

### 2.2. Experimental Design

In the current study, thirty male Wistar albino rats were utilized as subjects. The rats were divided into three groups using a random system. A normal control group of ten rats was given no medication at any point in the experiment. A single dose of APAP at 300 mg/kg was administered intraperitoneally (i.p.) to twenty rats. Randomly, these rats were divided into two groups. The I.P. administration of 1 mL/kg/day of 0.9% NaCl saline was administered to ten rats in Group 1 (APAP + Saline) for three days. The I.P. administration of 200 mg/kg/day of SIN was administered to ten rats in Group 2 (APAP + SIN) for three days.

**Figure 1:**
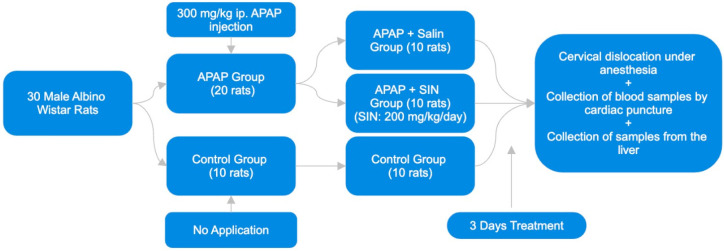
The flowchart of the study.

Ketamine (100 mg/kg, Ketasol, Richterpharma AG Austria) and xylazine (50 mg/kg, Rompun, Bayer, Germany) were the two components of the anesthesia that were administered to all of the animals at the end of the research project. The cervical dislocation method was used to put an end to the animals’ lives. In order to conduct biochemical analysis, blood samples were subsequently obtained through the use of cardiac puncture. The collection of liver samples followed this to conduct histopathological and biochemical examinations. The timetable and design of the study are shown in Figure 1.

### 2.3. Histopathological Studies of the Liver

Following the extraction of the liver, it was submerged in a solution of 10% formaldehyde in 0.1 M phosphate-buffered saline (PBS) for three days. With the help of hematoxylin and eosin, the liver sections, which were 4 μm in size and had been fixed with formalin, were stained. The Olympus C-5050 digital camera was mounted on the Olympus BX51 microscope (Shinjuku, Tokyo, Japan) to photograph each section. A computerized image analysis system (Image-Pro Express 1.4.5, Media Cybernetics, Inc., Rockville, MD, USA) was used to perform a morphological analysis on the liver. The evaluation was performed on ten microscopic fields for each section, with a magnification of forty times over. Knowledge of the study group among the observers conducting the evaluation was needed. A quantification was performed on the percentage of hepatocytes that had been damaged.

### 2.4. Determination of Plasma ALT Level

The plasma alanine aminotransferase (ALT) levels were assessed using a commercially available enzyme-linked immunosorbent assay (ELISA) kit from USCN Life Science Inc. (Wuhan, China).

### 2.5. Liver Biochemical Analysis

After sacrifice, the livers were immediately removed and kept at a temperature of −20 °C until they were analyzed biochemically. The liver was finely ground using a glass homogenizer in a solution of phosphate-buffered saline (pH 7.4), which was five times the volume of the liver. The resulting mixture was then separated by spinning at a force of 5000 times the acceleration due to gravity for 15 min. Subsequently, the liquid component containing the particles in suspension was collected, and the total protein content in the mixtures was quantified using Bradford’s method, with bovine serum albumin utilized as the benchmark [15].

The liquid portion was analyzed for the concentrations of SIRT1 and BMP-7 using commercially available ELISA kits specifically designed for rats. The measurements of all samples from each animal were conducted twice, adhering to the manufacturer’s guidelines. The absorbances were quantified using a microplate reader (MultiscanGo Laboratory Equipment, Portsmouth, NH, USA).

### 2.6. Assessment of Hepatic Lipid Peroxidation

The measurement of lipid peroxidation in tissue samples was performed by evaluating the concentrations of malondialdehyde (MDA) as thiobarbituric acid reactive substances (TBARS) [25]. Trichloroacetic acid and TBARS reagent were added to the tissue samples, then mixed and incubated at 100 °C for 60 min. After being cooled on ice, the samples were centrifugated at a speed of 3000 revolutions per minute for 20 min. The liquid portion above the sediment was subsequently assessed for absorbance at a wavelength of 535 nanometers. The MDA levels were quantified using the standard calibration curve with tetraethoxypropane and expressed in nmol/mg protein units.

### 2.7. Quantification of Hepatic Glutathione (GSH) Concentrations

The liver samples were analyzed for GSH concentration using spectrophotometry, precisely Ellman’s method [26]. Thiols react with 5,5′-dithiobis-(2-nitrobenzoic acid) (DTNB), creating a colored anion. The intensity of this anion is highest at a wavelength of 412 nm. The GSH levels were measured using the standard calibration curve and reported as nmol/mg protein.

### 2.8. Statistical Analysis

The data were evaluated in the IBM SPSS 15.0 statistical package program. The descriptive statistics for variables are presented as the mean ± standard error of the mean (SEM). The normal distribution of the data of numerical variables was evaluated with the Shapiro–Wilk normality test. In comparing two groups, if the data were normally distributed, the independent sample *t*-test was used. The Mann–Whitney U Test was used if the data were not normally distributed. A value of *p* < 0.05 was considered statistically significant.

## 3. Results

### 3.1. Analysis of Histopathology Evaluation

**Figure 2:**
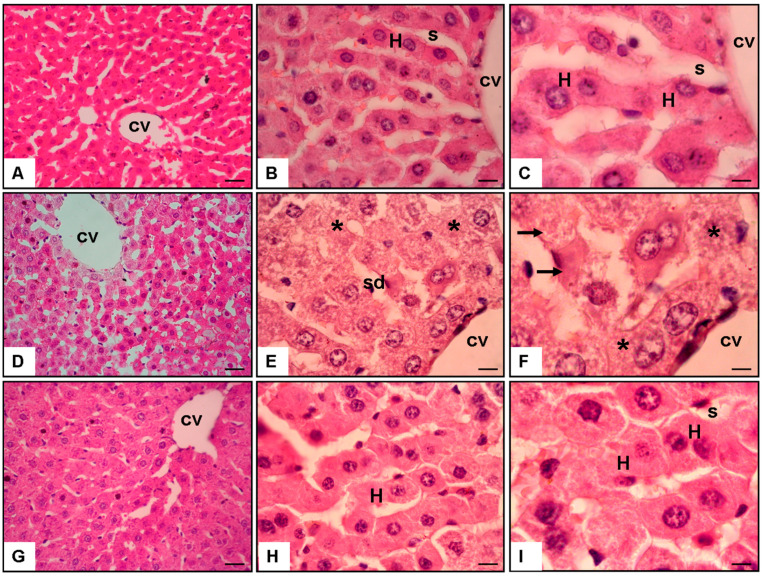
Hematoxylin and Eosine ((HE, scale bar = 100 μm)) staining of sections from rat liver (×20, ×40, ×100 magnification): (**A**–**C**) Normal Group liver, s: sinusoid, H: hepatocyte, and cv: central vein. (**D**–**F**) APAPand Saline Group liver, have cellular injury (asterisk), necrosis (arrow), and sinusoidal dilatation (sd) in centrilobular area of the liver. (**G**–**I**) APAPand 200 mg/kg/day of Sinomenine Group liver, no any cellular injury and necrosis in the centrilobular area of the liver.

The histological examination of liver sections stained with H and E provided detailed insights into the impact that SIN had on the architecture of the liver after the administration of APAP (Figure 2).

#### 3.1.1. Normal Group (Figure 2A–C)

Hepatocytes well preserved and arranged in rows were observed in the liver sections taken from the Normal Group. The architecture of the liver was found to be histologically intact. An open and regular pattern was observed in the sinusoids, indicating that the vascularization processes were normal. There was no evidence of inflammatory infiltrates, necrosis, or cellular abnormalities on the examined specimen. As shown in Figure 2, the hepatic parenchyma’s structural integrity was preserved, indicating a healthy liver phenotype.

#### 3.1.2. APAP + Saline Group (Figure 2D–F)

On the other hand, the liver sections obtained from the APAP and Saline Groups exhibited significant pathological alterations. In the centrilobular region of the liver, there was a clear indication of cellular injury, which was characterized by cellular swelling (asterisk), necrosis (arrow), and sinusoidal dilatation (sd). The presence of necrotic areas and the disruption of hepatocellular architecture were both indicators of severe liver damage that was caused by the toxicity of APAP. Additionally, the sinusoidal dilatation indicated a reduction in blood flow within the parenchyma of the liver (Figure 2).

#### 3.1.3. APAP + 200 mg/kg/day of SIN Group (Figure 2G–I)

The liver sections obtained from the group that received APAP and 200 mg/kg/day of SIN demonstrated a significant protective effect. Compared to the APAP and Saline Group, the centrilobular region exhibited no signs of cellular injury or necrosis. The hepatocellular architecture and the sinusoidal dilatation were significantly reduced, and the former appeared to be preserved. A protective environment was created within the liver tissue due to the administration of SIN, as indicated by this histopathological pattern (Figure 2). This indicated that the administration of SIN mitigated the deleterious effects of APAP.

The microscopic examination, which was carried out on ten fields in each section at a magnification of ×20 (Figure 2A–C), ×40 (Figure 2D–F), and ×100 (Figure 2G–I), provided support for the quantitative evaluation that was reported in the section on the results. This detailed examination of SIN to understand the morphological changes associated with drug-induced hepatotoxicity and potential recovery by therapeutic interventions provided additional evidence for the protective role played by SIN against APAP-induced liver toxicity. This further emphasizes the significance of histopathological analysis.

### 3.2. Biochemical Indicators of Liver Damage

#### 3.2.1. Alanine Aminotransferase (ALT)

In the Normal Group, the mean ALT level was 36.7 ± 1.9 U/L, indicative of baseline liver function. However, in the APAP and Saline Group, a substantial elevation in ALT levels was observed (183.5 ± 5.7 U/L, *p* < 0.001), signifying hepatocellular damage induced by Acetaminophen. Notably, the APAP and 200 mg/kg/day of SIN Group exhibited a significant reduction in ALT levels (62.7 ± 6.4 U/L, *p* < 0.05), suggesting a protective effect of SIN against APAP-induced liver injury (Table 1).

#### 3.2.2. Lipid Peroxidation (MDA Level)

The assessment of Malondialdehyde (MDA) levels, a marker of lipid peroxidation, revealed a baseline level of 1.25 ± 0.2 nmol/mg protein in the Normal Group. The APAP and Saline Group showed a marked increase in MDA levels (4.7 ± 0.1 nmol/mg protein, *p* < 0.001), indicating oxidative stress-induced damage. Conversely, the APAP and 200 mg/kg/day of SIN Group displayed a significant reduction in MDA levels (1.8 ± 0.2 nmol/mg protein, *p* < 0.05), highlighting SIN’s antioxidative properties and its role in mitigating lipid peroxidation (Table 1).

#### 3.2.3. Glutathione (GSH) Level

Evaluating Glutathione (GSH) levels, a crucial antioxidant, revealed a baseline level of 6.9 ± 0.2 nmol/mg protein in the Normal Group. The APAP and Saline Group showed a significant decrease in GSH levels (1.4 ± 0.2 nmol/mg protein, *p* < 0.001), indicating a compromised antioxidant defense. The administration of 200 mg/kg/day of SIN restored GSH levels significantly (4.7 ± 0.1 nmol/mg protein, *p* < 0.05), underscoring SIN’s hepatoprotective effect through the enhancement of the antioxidant capacity (Table 1).

#### 3.2.4. Sirtuin 1 (SIRT1) Level

The SIRT1 levels implicated in liver health and metabolic regulation were reduced in the APAP and Saline Group (1.4 ± 0.1 pg/g protein, *p* < 0.05) compared to the Normal Group (2.1 ± 0.2 pg/g protein). Although SIN treatment partially restored SIRT1 levels in the APAP and 200 mg/kg/day of SIN Group (1.9 ± 0.1 pg/g protein), the difference did not reach statistical significance (Table 1).

#### 3.2.5. Bone Morphogenetic Protein-7 (BMP-7) Level

The groups did not show any significant difference in the BMP-7 levels, indicating that SIN did not have a noticeable effect on this biochemical parameter in the context of APAP-induced liver damage (Table 1).

#### 3.2.6. Damaged Hepatocytes Percentage

The percentage of damaged hepatocytes significantly increased in the APAP and Saline Group (74.1 ± 6.9%, *p* < 0.001) compared to the Normal Group (2.7 ± 0.1%). The treatment with 200 mg/kg/day of SIN significantly attenuated hepatocyte damage (13.3 ± 2.8%, *p* < 0.05), further supporting the protective effect of SIN against APAP-induced hepatocellular injury (Table 1).

## 4. Discussion

When it comes to drug-induced hepatotoxicity, especially incidental cases, healthcare professionals in emergency departments and intensive care units are currently at a loss for what to do. Even though the liver is capable of regeneration, acute liver failure is still associated with a high mortality rate [27]. Understanding the factors that can either stimulate or inhibit liver regeneration is essential. Through the facilitation of liver regeneration, this knowledge has the potential to reduce mortality rates and the requirement for liver transplantation among individuals. The natural bioactive compound SIN was found to have a significant reparative effect on acute liver injury brought on by APAP, according to the findings of this study.

It is widely recognized that SOD confers protective properties against Reactive Oxygen Species (ROS), while MDA serves as a significant indicator of lipid peroxidation [28,29]. APAP administration increased ROS production, and the initiation of oxidative stress may result in hepatotoxicity [30]. The pathogenesis of liver diseases is significantly influenced by oxidative stress, marked by elevated MDA levels and reduced GSH levels [31]. In a study conducted by Li L [32], they describe that SIN exhibits a protective effect on rat cardiomyocytes by reducing the content of MDA and enhancing the activity of SOD in response to a free radical-induced injury. On the other hand, Ramazi et al. [33] found that 50 mg/kg of SIN effectively decreases the intensity of seizures and mitigates oxidative stress, inflammation, and apoptosis in the hippocampus of rats with temporal lobe epilepsy. Similarly, in this study, the results indicate that the administration of SIN resulted in a reduction of MDA levels dependent on the dose administered and an increase in GSH levels that was also dose-dependent. The cohort administered with a dose of 200 mg/kg of SIN demonstrated the lowest MDA levels and the most excellent GSH levels, suggesting SIN’s robust antioxidant characteristics.

This study is a constructive study on the inhibition and recovery of hepatocyte damage by SIN. Both in histological sections and in percentage terms, the study shows that it strongly reverses hepatocyte damage. Similarly, Feng et al. [34] describe that SIN protects the liver by stopping hepatocyte apoptosis and lowering inflammation. This keeps liver cells and sinusoidal endothelial cells safe from cold ischemia/reperfusion injury in rats getting an orthotopic liver transplant.

APAP overdose produces the highly reactive metabolite N-acetyl-p-benzoquinone imine. This metabolite causes GSH depletion, oxidative stress, and mitochondrial dysfunction. It causes immune response-mediated damage to the liver and severe hepatocyte necrosis [19,35]. There are various studies in liver transplant and hepatocellular carcinoma models for evaluating the immunosuppressive effect of SIN [34,36,37]. The immunosuppressive property of sinomenin inhibits the hepatotoxicity process in this cascade.

In a study conducted by Chen et al. [38], the APAP toxicity model was studied in 25, 50, and 100 mg/kg doses, and it was found that the anti-inflammatory and antioxidant capacity increased as the dose increased. Furthermore, in this study, 200 mg/kg was tested for the first time using the same experimental model and an effect parallel to the dose-response curve in the other study was found.

The human protein SIRT-1 is of great importance in the processes of inflammation and neurodegeneration [39]. In the study of El-Sheikh et al. [40], they observe that Resveratrol can reduce radiation-induced liver damage by reducing apoptosis and inflammation through the modulation of SIRT1 activity. Moreover, Biel T [41] found that SIRT1 plays a crucial role in preventing ischemic liver injury by suppressing defective autophagy, mitochondrial dysfunction, and hepatocyte death, with its activation dependent on mitofusin-2. Recent studies emphasize the therapeutic role of SIRT1 in the treatment of drug-induced liver injury (DILI) with the alleviation of apoptosis [42,43]. Therefore, the clinical application of SIRT1 activators (Resveratrol, Quercetin, Melatonin) is the default therapeutic approach against DILI. A statistically significant change was noticed in the SIN + APAP Group compared to the APAP + Saline Group in SIRT-1 levels. This shows that SIN is an activator of SIRT-1 for preventing liver injury.

Alternatively, Sugimoto et al. suggested that circulating BMP-7 is a potent regulator of hepatocyte health and an internal regulator of hepatocyte function. Given that this effect is likely facilitated by circulating BMP-7 in the kidney and bone, the researchers concluded that BMP-7 functions as a hormone in the liver [24]. A study conducted by Wang L et al. [44] found that BMP-7 achieves the attenuation of liver fibrosis through the regulation of the epidermal growth factor receptor. In our study, we observe that with the admission of SIN, the liver BMP-7 increased; these results suggest that although BMP-7 is not secreted from the liver, BMP-7 receptors in the liver bind to BMP-7 in case of damage, and thus, it is beneficial in the process of liver healing and preventing damage. In another study, Cao H. et al. [45] observed that BMP-7 expression in the liver increases with liver inflammation and fibrosis severity, suggesting its potential role as an anti-inflammatory and anti-fibrogenic agent in chronic hepatitis. In a study by Zou et al. [46], the upregulation of BMP-7 in the liver during the initial phase of liver fibrosis primarily functions to inhibit fibrosis.

Animal models and patients with liver fibrosis have also provided evidence of BMP-7’s antifibrotic effects on the liver. The in vitro experiments validated that elevated BMP-7 effectively inhibits HSC activation and collagen formation, thereby exerting its anti-fibrosis function. Within the mouse model, the expression level of BMP-7 initially exhibited an increase, subsequently transitioning into a decrease as liver fibrosis advanced. The analysis of BMP-7 expression in samples from patients with liver fibrosis and cirrhosis revealed a consistent pattern of initially increasing expression, followed by a subsequent decrease. The diminished expression of BMP-7 in the advanced stage was strongly linked to the upregulation of TGF-β1 expression. It is hypothesized that TGF-β1 has a negative effect on the expression of BMP-7, but this inhibition occurs only within a specific range of dosages. Simply put, large amounts of BMP-7 may counteract TGF signaling.

All these results show that Sinomenine has a restorative effect and facilitates the reparation of the liver. Moreover, the histopathologic evidence after SIN admission is obvious, and the results are supported with hepatocyte damage percents.

The limitations of this study are that it is an animal experiment and the ethical effects on humans may differ. Another limitation is that the other MSCs should be evaluated across a broader spectrum.

## 5. Conclusions

This study provides evidence that Sinomenine treatment reduces APAP-induced liver injury. Sinomenine may be beneficial in liver diseases with similar pathology. Future studies should examine the long-term benefits, optimal dose, and side effects of Sinomenine therapy for clinical safety and efficacy.

## Figures and Tables

**Table 1:** Biochemical indicators of liver damage.

	Normal Group	APAP + Saline	APAP + 200 mg/kg/day of Sinomenine
ALT (U/L)	36.7 ± 1.9	183.5 ± 5.7 **	62.7 ± 6.4 ^##^
Liver MDA Level (nmol/mg protein)	1.25 ± 0.2	4.7 ± 0.1 *	1.8 ± 0.2 ^##^
Liver GSH Level (nmol/mg protein)	6.9 ± 0.2	1.4 ± 0.2 **	4.7 ± 0.1 ^##^
Liver SIRT1 Level (pg/g protein)	2.1 ± 0.2	1.4 ± 0.1 *	1.9 ± 0.1 ^#^
Liver BMP-7 Level (pg/g protein)	55.6 ± 2.8	50.8 ± 4.4	73.6 ± 3.5 ^#^
Damaged hepatocytes (percent)	2.7 ± 0.1	74.1 ± 6.9 **	13.3 ± 2.8 ^##^

Data are expressed as mean ± SEM. * *p* < 0.05, ** *p* < 0.001(different from control), ^#^ *p* < 0.05, ^##^ *p* < 0.001 (different from PST + Saline).

## Data Availability

Data may be shared with an appropriate justification if requested.
